# Burnout among community service doctors in South Africa

**DOI:** 10.4102/phcfm.v16i1.4436

**Published:** 2024-05-15

**Authors:** Gemma M. Purbrick, Tejil Morar, Jasmin Kooverjee

**Affiliations:** 1 Department of Psychiatry, Faculty of Health Sciences, University of the Witwatersrand, Johannesburg, South Africa

**Keywords:** burnout, junior doctors, community service, South Africa, MBI

## Abstract

**Background:**

Burnout in doctors is an important issue with far-reaching consequences. Community service doctors may be particularly vulnerable because of their specific roles (rural settings, junior positions and reduced supervision).

**Aim:**

This study aimed to determine the prevalence of burnout among community service doctors in South Africa (SA), the potential contributory and protective factors and the consequences thereof.

**Setting:**

This was a national study of community service doctors in SA.

**Methods:**

A quantitative, descriptive cross-sectional study was performed. The Maslach Burnout Inventory was used to measure burnout. The online questionnaire also included demographic data, workplace and individual characteristics.

**Results:**

Of the 208 community service doctors analysed, 89% and 94% had high emotional exhaustion and depersonalisation, respectively, while 97% had a low personal accomplishment. Mental illness, financial difficulties, unmanageable volume of patients and female gender were found to be potential contributory factors. Having manageable patient volumes, satisfaction with their decision to study medicine, talking to colleagues and feeling supported by healthcare facility management were among the significant potential protective factors. Significant potential consequences of burnout included: leaving the government sector, ever being diagnosed with a mental illness, using alcohol as a coping mechanism and possible current major depression.

**Conclusion:**

Burnout among community service doctors in SA is highly prevalent with significant potential consequences. There are a number of modifiable possible contributory and protective factors identified that may be targets for mental health interventions.

**Contribution:**

Healthcare burnout research is lacking in the African and specifically SA context. This void includes community service doctors.

## Introduction

Burnout in doctors is an important issue with significant prevalence and far-reaching consequences.^1^ Although there has been much research on burnout among junior doctors globally, research is lacking when it comes to the African, and specifically, the South African context. This void includes community service doctors whose specific role may influence vulnerability to burnout as they are required to work as independent medical practitioners for the first time with minimal, if any, supervision and as a result increased responsibility.^2,3^ Community service is a compulsory remunerated 1-year initiative for medical doctors, following graduation from medical school and a 2-year internship programme. It was introduced post-Apartheid in line with government’s movement towards decentralised, or primary, healthcare. It is designed to further practical clinical experience and learning, as well as to reduce staff shortages in under-resourced healthcare facilities, usually in rural areas.^2^

Burnout is characterised by three components: feelings of emotional exhaustion (EE), depersonalisation (DP) and reduced feelings of personal accomplishment (PA), which can be measured using the Maslach Burnout Inventory (MBI).^4^ Depersonalisation is described as an emotional detachment from work, reduced feelings of PA include poor self-evaluation and sense of achievement, and EE entails loss of enjoyment for work.^4^ Although not a Diagnostic and Statistical Manual of Mental Disorders’ Text Revision-5 (DSM-TR-5) diagnosis, burnout has been included in the International Classification of Diseases-11 (ICD-11) under code Z73.0.^5^ The prevalence rates of burnout vary in the literature.^6^ The prevalence of burnout among the general population in high-income countries is reported to be between 13% and 27%.^7^ In comparison, the rate among healthcare workers has generally been higher – up to 70% in some studies.^7^ Studies of burnout in sub-Saharan Africa indicate significant prevalence as well.^7,8,9,10^ A 2021 study on doctors in rural KwaZulu-Natal (KZN) found that 85.2% community service doctors were burnt out in their sample.^11^

Several factors have been shown to contribute to burnout in doctors. Younger doctors have been found to be more susceptible to burnout than their older peers.^3,12^ A 2013 South African study on rural doctors in the Western Cape province found that junior rank is associated with burnout.^9^ This was corroborated in a 2020 study on doctors in the KZN province of South Africa (SA) where an association particularly with lower levels of PA and higher levels of DP was found.^9^ According to a 2020 systematic review on burnout in trainee physicians, those reporting a negative workplace environment were twice as likely to report burnout.^13^ Heavy workloads, dysfunctional work conditions, poor work-life balance and patient care concerns were examples of such negative workplaces.^14^ In a South African study on doctors working in Cape Town’s community healthcare clinics and district hospitals, the above-mentioned factors were also noticed to be important with number of hours worked, work load and working conditions being ranked as the top three most important.^10^

If not recognised, burnout in doctors has several consequences for mental health, overall physical health and quality of life.^10^ In a South African study of doctors in KZN, burnout was significantly associated with both depression (*p* < 0.01) and anxiety (*p* < 0.01).^8^ For patients, there are aftereffects of doctor burnout. Self-reported low-quality patient care, higher patient mortality rates and less time performing clinical care duties were significantly associated with burnout in a study on physicians in the United States between 2008 and 2014.^15^ In a 2008 study of American surgeons, medical errors are more frequent among those with burnout.^16^ The reduction in empathy as a consequence of burnout negatively impacts the quality of patient experience.^14^ Furthermore, and perhaps of particular concern in a resource-limited healthcare setting such as SA, is the monetary cost of doctor burnout to the system itself as a result of legal cases (as a result of poor patient care) and long term employee absences (because of mental health diagnoses).^17^ Retention of doctors is also a concern. Those who are burnt out are more likely to leave the profession.^11^

To mitigate the negative consequences of burnout, protective factors need to be explored and strengthened. A 2020 systematic review found that strong, stable interpersonal relationships are associated with less reported burnout and those with adequate senior supervision are less likely to report burnout.^14^ Support from colleagues and working as a team play a significant role, and support from hospital management is significantly associated with lower burnout rates.^3,4,14^ Personal factors, such as timely help-seeking behaviour, coping skills and finding meaning in one’s work are associated with less risk of burnout.^1^

Occupational mindfulness programmes and organisational mental health initiatives are interventions that have been found to reduce rates of burnout.^4^ There is a paucity of published data on doctors’ awareness of their own burnout. However, a 2021 study on pharmacists in Canada, found that of those who objectively met criteria for burnout, 23% said they subjectively had no burnout.^18^

The aim of this study was to explore burnout in community service doctors in SA. The objectives included its prevalence and associated protective, contributory and consequential factors (including possible depression). Identifying potential protective and contributory factors may aid to better serve doctors, patients and the community at large. It is the first step to inform appropriate interventions, which optimise the mental health of junior doctors and their overall functioning as well as improve patient care.^7^

## Methods

### Study design

This study employed a descriptive cross-sectional study design.

### Study setting

The study takes place against SA’s background of an under-resourced public health system. The community service doctor is a junior doctor who has just completed a 2 year period of internship, under supervision, to enhance his or her clinical knowledge. They are now considered to be able to work with only minimal, and often no, supervision but are not yet allowed to register as independent practitioners. Community service is a compulsory 1 year training post designed to further practical clinical experience and learning, as well as address staff shortages in under-resourced healthcare facilities, usually in rural areas. It is a remunerated year.^2^

### Study population

All doctors in their community service year in SA (national study) were included in the study. Doctors who were not currently community service doctors registered in South Africa were excluded.

### Sample size calculation

The estimated population of community service doctors in South Africa is 2000 (between 1708 and 2252).^19^ The sample size was calculated using an estimated prevalence of 85.2%, from a 2021 KZN study that included community service doctors,^11^ a confidence level of 95%, and precision of 5%. A finite sample size correction was applied as the population size was under 2000 people, resulting in a minimum sample size of 177.

### Approach to sampling

Attempts were made to contact the Health Professions Council of South Africa (HPCSA) to assist with distribution, but to no avail. The South African Medical Association (SAMA), the largest non-statutory professional association for public and private sector medical practitioners,^20^ then assisted with distributing the invitation. Social media platforms were also utilised to access potential participants who may not be registered with SAMA. Convenience sampling was therefore used as all respondents were self-selected in a non-probability sampling method.

### Data collection

The MBI Human Services Survey for Medical Personnel (MBI-HSS [MP]) was used to assess burnout and the PHQ-9 was used to screen for depression in this study. There are a number of other scales used to measure burnout including the Oldenburg Burnout Inventory, Single Item Burnout Measure and the Copenhagen Burnout Inventory. The MBI, however, is the most commonly used having shown to have the highest construct validity among healthcare practitioners.^4^ The MBI-HSS (MP) is a modification of the MBI adapted for healthcare providers. It comprises 22 questions divided into three sections that correlate with the three different components of burnout described above (EE, DP and PA).^4^ Based on responses to these questions, individuals are classified as having either a low, moderate or high level of burnout^21^ (Table 1). These are based on the cut-off scores used in the third edition of the MBI manual, which correlate to scores predominantly used in burnout research.^21^ A person who had high EE, DP, and low PA was considered as having ‘burnout syndrome’.

**TABLE 1:** Classification of burnout.

Category	Low	Moderate	High
EE (9 questions)	≤ 18	19–26	≥ 27
DP (5 questions)	≤ 5	6–9	≥ 10
PA (8 questions)	≥ 40	39–34	≤ 33

*Source*: Doulougeri K, Georganta K, Montgomery A. ‘Diagnosing’ burnout among healthcare professionals: Can we find consensus? *Cogent Med*. 2016 Oct 17;3(1):1. https://doi.org/10.1080/2331205X.2016.1237605

EE, emotional exhaustion; DP, depersonalisation; PA, personal accomplishment.

Permission to use an electronic version of the MBI-HSS (MP) was acquired from www.mindgarden.com. The Patient Health Questionnaire-9 (PHQ-9) is the most commonly used screening tool for depression.^22^ It consists of nine questions and is designed to be used in primary care and other settings to screen for depression. A cut-off score of 10 in the PHQ-9 was used to identify possible major depression.^22^ This score has a sensitivity of 0.88 and a specificity of 0.85.^22^

An electronic REDCap questionnaire was distributed to community service doctors in SA via SAMA and social media platforms, that is South African Medical Doctors Community Service Facebook Group and WhatsApp from October to December 2022. All doctors in their community service year in SA who had access to the questionnaire during the data collection period were invited to participate in the study. The questionnaire consisted of four sections. The first section contained sociodemographic questions, the second section was the MBI-HSS (MP), the third section was the PHQ-9 and the final section addressed potential factors associated with burnout based on a literature review. The final question asked to what degree the participants subjectively believed they were burnt out. In total, the survey contained 40 questions, including 10 Likert scale questions, in addition to the 9 questions for the PHQ-9 and the 22 questions of the MBI-HSS. The questionnaire was developed by the researchers based on the review of the literature (Table 2).

**TABLE 2:** Potential factors associated with burnout.

Contributory factors	Protective factors	Consequential factors
**Workplace factors**		
Heavy workloads^13^	Adequate senior supervision^13^	More likely to be absent^24^
Dysfunctional work conditions^13^	Support from colleagues^2,7,13^	Reduced skills retention^3^
Poor work-life balance^13^	Support from hospital management^7^	Less time performing clinical care duties^15^
Patient care concerns^13^	-	-
Shifts > 12 hours^23^	-	-
Emergency and/or surgical specialities^14^	-	-
A lack of resources^8^	-	-
Inadequate supervision^8,13^	-	-
Staff shortages^7^	-	-
Demanding workloads^7^	-	-
Poor career satisfaction^7,13^	-	-
**Individual factors**		
Female gender^13^	Timely help seeking behaviour^1^	-
Younger age^7,13^	Coping skills^1^	-
Self-efficacy^13^	Finding meaning in one’s work^1,7^	-
Perceived physical and mental well-being^13^	Strong, stable interpersonal relationships^7,13^	-
Financial stressors^13^	-	-
Junior rank^8,13^	-	-
**For patients**		
-	-	Low-quality patient care^15^
-	-	Higher mortality rates^15^
-	-	More frequent medical errors^16^
-	-	Quality of patient experience^17^
**For doctors**		
-	-	Depression^9,25,26^
-	-	Anxiety^8^
-	-	Hypercholesterolaemia^24^
-	-	Type 2 diabetes^24^

Note: Please see the full reference list of the article, Purbrick GM, Morar T, Kooverjee, J. Burnout among community service doctors in South Africa. Afr J Prm Health Care Fam Med. 2024;16(1), a4436. https://doi.org/10.4102/phcfm.v16i1.4436, for more information.

### Statistical analysis

The analysis was carried out using the latest version of Rstudio, a statistical program. A *p*-value of < 0.05 was used to indicate statistical significance. Descriptive statistics were used for the EE, DP and PA components of burnout and then classified according to Table 1.

Descriptive statistics reporting the natural frequency (*n*) and proportion (%) of responses are tabulated and compared with having a high EE, high DP and low PA. Univariate logistic regression was used to calculate the Prevalence Odds Ratio (OR) of association on each response variable and the outcome variable (high burnout scores and burnout syndrome). The OR point estimate, 95% confidence intervals and associated *p*-values are tabulated (Table 3).

**TABLE 3:** Other *p*otential *a*ssociated *f*actors to the three burnout components.

Characteristic variable	High EE			High DP			Low PA (< 32)		
	OR	95% CI	*P*	OR	95% CI	*P*	OR	95% CI	*P*
**Age (years)**	1.38	1.00, 2.11	0.046	0.85	0.69, 1.09	0.2	0.80	0.63, 1.07	0.12
**Experienced gender**									
Male	–	–		–	–		–	–	
Female	1.97	0.77, 4.79	0.2	0.70	0.13, 2.48	0.6	4.55	1.07, 21.0	0.041
**Number of children**									
0	–	–		–	–		–	–	
1	0.91	0.20, 8.74	>0.9	0.24	0.06, 1.35	0.10	0.79	0.08, 106	0.9
2	0.39	0.02, 57.8	0.6	0.19	0.01, 28.1	0.4	0.10	0.00, 15.7	0.3
> 2	0.65	0.05, 91.1	0.8	0.31	0.02, 44.2	0.5	0.03	0.00, 0.47	0.016
True	0.52	0.19, 1.28	0.2	0.31	0.06, 1.07	0.065	0.67	0.12, 2.86	0.6
**Coping mechanisms**									
Meditation	0.51	0.20, 1.45	0.2	0.40	0.13, 1.45	0.2	0.44	0.10, 2.51	0.3
Alcohol	1.06	0.42, 2.95	> 0.9	11.5	1.48, 1.479	0.013	0.29	0.06, 1.22	0.089
Cannabis	0.51	0.14, 2.80	0.4	1.84	0.22, 240	0.7	0.26	0.05, 2.66	0.2
Illicit illegal drugs	0.46	0.09, 4.60	0.4	0.93	0.10, 123	>0.9	0.12	0.02, 1.30	0.075
Therapy	1.82	0.55, 9.36	0.4	0.57	0.17, 2.38	0.4	0.42	0.10, 2.43	0.3
Over the counter medication	3.97	0.50, 514	0.2	2.16	0.26, 281	0.6	0.15	0.03, 0.87	0.037
Prescription medication	2.38	0.57, 22.0	0.3	0.66	0.18, 3.53	0.6	0.30	0.07, 1.76	0.2
Support groups	1.43	0.15, 190	0.8	0.78	0.08, 104	0.9	0.42	0.04, 57.0	0.6
Talking to friends family	0.81	0.20, 2.40	0.7	1.05	0.20, 3.80	>0.9	2.29	0.40, 9.95	0.3
Talking to colleagues	1.41	0.58, 3.70	0.5	0.69	0.23, 2.13	0.5	0.13	0.01, 0.64	0.011
Talking to seniors	5.82	0.75, 749	0.11	3.15	0.39, 409	0.3	0.46	0.09, 4.61	0.4
**Likert scales**									
**I feel supported by hospital management**									
Neutral	–	–		–	–		–	–	
Strongly disagree	0.85	0.24, 2.60	0.8	2.67	0.72, 10.4	0.14	0.17	0.00, 1.60	0.14
Disagree	0.86	0.22, 3.03	0.8	2.11	0.53, 9.58	0.3	0.41	0.00, 7.95	0.6
Agree	0.33	0.07, 1.65	0.2	1.13	0.20, 11.8	0.9	0.30	0.00, 56.0	0.6
Strongly agree	0.33	0.01, 50.4	0.5	0.41	0.02, 62.5	0.6	0.00	0.00, 0.14	0.003
**The volume of patients I am expected to see is manageable**									
Neutral	–	–		–	–		–	–	
Strongly disagree	9.87	1.07, 1.31	0.042	6.03	0.56, 819	0.2	0.33	0.03, 2.10	0.2
Disagree	1.16	0.33, 4.16	0.8	2.80	0.44, 29.5	0.3	0.48	0.05, 3.01	0.4
Agree	0.29	0.09, 0.86	0.025	0.37	0.08, 1.39	0.14	2.31	0.12, 341	0.6
Strongly agree	0.43	0.09, 2.68	0.3	0.17	0.03, 0.91	0.040	0.66	0.03, 98.4	0.8
**When not on call I am required to stay at work after the recommended end of work time 16:00**									
Neutral	–	–		–	–		–	–	
Strongly disagree	0.32	0.06, 1.25	0.11	0.13	0.00, 1.24	0.081	2.14	0.27, 24.1	0.5
Disagree	0.40	0.07, 1.61	0.2	0.09	0.00, 0.80	0.027	1.34	0.20, 9.05	0.8
Agree	0.77	0.13, 3.71	0.8	0.19	0.00, 2.07	0.2	2.46	0.31, 27.8	0.4
Strongly agree	0.61	0.09, 4.19	0.6	0.63	0.00, 118	0.8	1.06	0.13, 12.1	> 0.9
**Self-described current burnout status**									
I am not burnout	–	–		–	–		–	–	
Low burnout	15.1	3.95, 69.4	< 0.001	1.25	0.20, 6.36	0.8	0.72	0.00, 14.2	0.8
Average burnout	28.8	8.39, 114	< 0.001	2.84	0.47, 13.1	0.2	1.91	0.01, 37.4	0.7
High Burnout	49.8	11.3, 315	< 0.001	4.10	0.59, 28.8	0.14	0.31	0.00, 2.99	0.4

EE, Emotional Exhaustion; DP, Depersonalisation; PA, Personal Accomplishment.

### Ethical considerations

The questionnaire was stored on an encrypted REDCap database and only the researcher and supervisors had access to this database. The questionnaire began with a *Protection of Personal Information Act* (POPIA)-compliant information page. Participants were able to exit the questionnaire and retract informed consent at any point during the survey. Participants in the study remained anonymous. A distress protocol was included in the introduction section as well as at the end of the questionnaire should participants have felt that they required mental health support after completing the survey.

Ethical approval was provided by the University of the Witwatersrand (Wits), Human Research Ethics Committee (HREC) (medical). Reference number: M220532.

## Results

### Demographics of the sample population

Of the estimated 2000 community service doctors employed during this period, 228 responses were received. However, 20 responses were excluded because of excessive missing information or the respondent reporting not being a community service doctor. Thus, 208 questionnaires were used for data analysis.

The median age of the population was 27 years old (IQR 26–28). Participants in a relationship accounted for 36% of the population and 93% had no children. Gauteng province represented the location of most participants (27%) followed by Western Cape (13%). Fifty-two per cent were located outside of metropolitan areas with the majority working in district hospitals (44%). Family and emergency medicine were the most common specialities at 38% and 13%, respectively (Table 4).

**TABLE 4:** Demographics and job characteristics of the sample population (*N* = 208).

Characteristic	Overall	
	*n*	%
**Experienced gender**		
Male	48	23
Female	160	77
**Relationship status**		
Divorced and/or separated	1	0.5
In a relationship	75	36
Married	61	29
Single	71	34
**Number of children**		
0	194	93
1	11	5.3
2	1	0.5
> 2	2	1.0
**Province of work**		
Eastern Cape	21	10
Free State	14	6.7
Gauteng	57	27
KwaZulu-Natal	13	6.3
Limpopo	17	8.2
Mpumalanga	30	14
North West	20	9.6
Northern Cape	8	3.8
Western Cape	28	13
**Facility location**		
Rural	81	39
Metropolitan	100	48
Other	27	13
**Facility type**		
Primary Healthcare (Clinics)	32	15
District Hospital	91	44
Regional Hospital	43	21
Tertiary or Quaternary (Academic) Hospital	42	20
**Current department**		
Anaesthesia	9	4.3
Ear, Nose and Throat (ENT)	1	0.5
Emergency Medicine	32	15
Family Medicine (Clinics, General OPDs, General Male and/or Female Wards)	79	38
General Surgery	10	4.8
Internal Medicine	18	8.7
National Health Laboratory Service	2	1.0
Neurosurgery	2	1.0
Obstetrics and Gynaecology	13	6.3
Ophthalmology	5	2.4
Orthopaedics	3	1.4
Other	12	5.8
Paediatrics	16	7.7
Psychiatry	6	2.9

Note: Age (years): OR 27.00, 95%CI 27.00, 28.00.

### Prevalence of burnout

**FIGURE 1:**
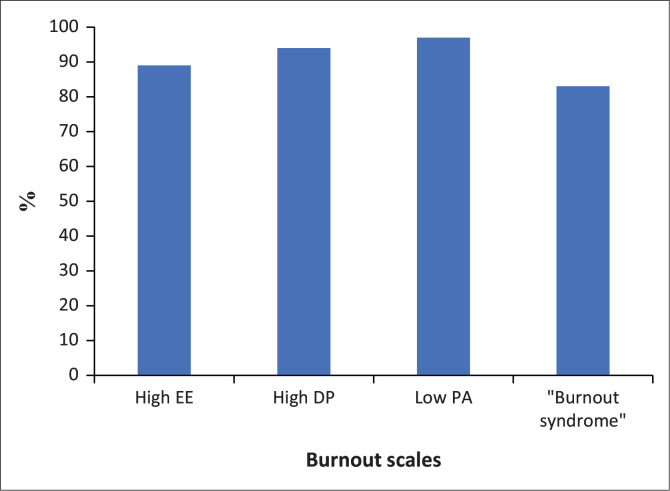
Burnout prevalence among sample population.

Of the 208 participants, 89% had high EE (*N* = 185), 94% (*N* = 195) had high DP, while 97% (*N* = 201) had a low sense of PA (Figure 1). Higher EE and DP scores and lower PA score correlate to increased experience of burnout. These results are based on the classification system depicted in Table 1. Furthermore, 83% had ‘burnout syndrome’ – where a participant had both high DP and EE as well as low PA (Figure 1). Of those with burnout syndrome, 69% felt they had ‘no to average’ burnout (*p* < 0.001). No participant had low levels of burnout on all three subscales (i.e. low EE, DP and high PA).

### Possible contributory factors

The odds of having a high EE are significantly associated with ever being diagnosed with a mental illness (OR 4.25, 95%CI [1.31,21.6]), financial difficulty (OR 5.14, 95%CI [1.27,47.0]), those who strongly disagree that the volume of patients they are expected to see is manageable (OR 9.87 95%CI [1.07, 1.309]). The odds of having high DP are significantly associated with doing > 30 hours of actual overtime per week (OR 39.9 95%CI [1.80,6.298,41]), individual calls lasting 24–30 hours (OR 7.69, 95%CI [1.15,13.6]).

The odds of having a low PA are significantly associated with female experienced gender (OR 4.55, 95%CI [1.07, 21.0]) (Table 3).

### Possible protective factors

The odds of having a low EE are significantly associated with working at a district hospital (OR 3.86, 95%CI [1.14,13.6]), agreeing to have a manageable volume of patients (OR 0.29, 95%CI [0.09, 0.86]). The odds of having a low DP are significantly associated with those who strongly agree that they are satisfied with their decision to study medicine and become a doctor (OR 0.11 95%CI [0.01, 0.55]), strongly agree to having a manageable volume of patients expected to see (OR 0.17, 95%CI [0.03, 0.91]), disagree that they are required to stay at work after the recommended end of work time (OR 0.09, 95%CI [0.00,0.80]). The odds of having a high PA are significantly associated with having >2 children (OR 0.03, 95%CI [0.00,0.47]), taking over the counter (OTC) medication (OR 0.15, 95%CI [0.03,0.87]), talking to colleagues as a coping mechanism (OR 0.13, 95%CI [0.09, 4.61]) and strongly agreeing that they feel supported by hospital management (OR 0.00, 95%CI [0.00, 0.14]) (Table 3).

### Possible consequences associated with burnout

The odds of having a high EE are significantly associated with leaving the government sector (private sector, different field, sabbatical) (OR 3.38, 95%CI [1.37, 9.35]) and ever being diagnosed with a mental illness (OR 4.25, 95%CI [1.31,21.6]). The odds of having a high DP are significantly associated with leaving the government sector (for the private sector, a different field or sabbatical) (OR 3.57, 95%CI [1.12, 14.5]), using alcohol as a coping mechanism (OR 11.5, 95%CI [1.48, 1.479]) (Table 3). There were no significant associations with having a low PA.

Sufficient basic resources, adequate senior support, good social support system at and outside of work, and staff shortages were also variables that were tested. However, no significant relationship to burnout was found.

### Depression

Forty-nine per cent of respondents had possible major depression using the PHQ-9. The proportion of respondents with a high EE score is higher in those with potential depression (98%) (95%CI [93,100]) compared with those without potential depression (79%) (95%CI [70,87]), *p*-value < 0.001. The same is true for DP with 97% having potential depression 95%CI (91,100) versus 90% without (95%CI [82,95]), *p*-value 0.017 (Table 3).

The odds of having high EE were 11 times higher for those who were potentially depressed compared with those who were not potentially depressed (95%CI 75.9,77.798) (Table 3).

## Discussion

### Summary of key findings

The key finding of this study is that the prevalence of burnout in community service doctors was higher than the burnout prevalence of doctors internationally.^6^ Eighty-nine per cent of respondents had high EE, 94% had high DP while 97% had a low sense of PA and 83% had both high EE and DP as well as low PA (i.e. all three). There was no participant in this study who had low levels of burnout on all three subscales. Participants were not aware of their burnt-out status.

### Discussion of key findings

There were significantly more female respondents than male respondents. This may be because of transformation policies when recruiting medical students becoming effective. However, it may also be because of a respondent bias. Of the individual factors, female gender had significantly higher odds of burnout in this study. This is in agreement with a 2020 systematic review on physician burnout.^13^ However, our study did not find any other demographic factors to significantly correlate with burnout.

This may be because the age range was too small to show a significant difference as a large proportion of the sample was of the same age. A 2019 systematic review of studies conducted on burnout among healthcare providers in sub-Saharan Africa found between 9.1% and 65.2% of doctors had high EE, 33.37% and 85.1% had high DP and 25% and 91% experienced low PA.^7^ The countries studied here were Nigeria, Ethiopia, SA and Ghana. The results of this study were higher or towards the upper end of these values. This could be because of the junior rank (less experience of community service doctors often with less supervision).

In a 2020 study of 150 doctors in the South African province of KwaZulu-Natal (KZN), 59% were found to be burnt out based on either high EE or DP scores.^8^ A total of 48.7%, 45.3% and 22% had high EE, DP and low PA, respectively. These rates of burnout are lower than this study. The aforementioned study did not include community service doctors but did include interns, medical officers (a rank above community service doctors), registrars and specialists. They also found that level of experience results in less burnout, potentially explaining the differences. Their data are below the expected level for SA and the authors postulate that this may be because other studies were performed on more junior doctors.^8^ A 2021 study on doctors in rural KZN found that 68.5% were burnt out based on either high EE or DP. However, of the study’s subgroup of community service doctors, 85.2% were found to be burnt out. The results of this study were slightly higher.^11^

A 2023 study on interns, medical officers, registrars (‘residents’) and consultants at a tertiary hospital in Johannesburg, found that 46.2% of these doctors screened positive for burnout (based on high EE and high DP), with 60.9% scoring high on EE, 59.9% scoring high on DP and 55.4% scoring low on PA.^12^ Again, this study found that age was an associated factor with burnout, explaining the discrepancy between this study’s results as the ages of the target populations are different.

This study found protective factors in keeping with those identified in a systematic review of physicians and burnout, including workplace and individual factors.^13^ Having > 2 children was, however, a new finding. Perhaps having to deal with the responsibility of multiple children could prepare community service doctors for similar work challenges or assist in providing perspective in work-life balance.

In this study, possible contributory factors to burnout included, participants who identify as female, those who had longer hours of overtime, financial difficulty, those who felt they had an unmanageable patient load as well as those who felt they did not have a good work-life balance. A consistent finding in this study and a number of other studies was the association of burnout with a negative workplace environment (Table 2).

A 2018 meta-analysis on medical residents found that those in emergency or surgical specialities had a higher level of burnout.^14^ However, this study did not find significant correlation between the department and increased levels of burnout. This may be because of the fact that community service doctors often rotate through disciplines during the year, unlike residents who remain in a certain department.

Possible major depression, taking OTC medication, being diagnosed with a mental illness, using alcohol as a coping mechanism and planning to leave the government sector were all potential consequences that were found in this study. The latter is a particularly detrimental consequence for the South African state sector, where a shortage of trained doctors exists.^27^ In the literature, on a societal level, burnout may have an impact on skills retention, as those who are burnt out are more likely to leave the profession.^1^ A 2009 South African study found that South African community service doctors who had a high level of professional development were twice as likely to remain in rural areas impacting skills retention.^28^ The association of using alcohol with burnout may indicate that participants are self-medicating symptoms of burnout with alcohol.

In the above-mentioned 2023 study conducted in a tertiary hospital in Johannesburg, 53.73% screened positive for major depression on the PHQ-9 while 49% screened positive for major depression in this study’s participants. The correlation between burnout and possible depression was significant with potential major depression positively correlating to EE and DP, suggesting that those who are burnt out are more likely to be depressed. A number of both local and international studies found an association between depression and burnout in doctors.^9,12,15,25^ However, a study of Austrian physicians did not.^29^

Of those with burnout syndrome (83%), 69% felt that they had ‘no to average’ burnout (*p* < 0.001). This suggests that community service doctors were not able to correctly assess their own burnout level, which may impact on appropriate health-seeking behaviour. There is a dearth of research into doctors’ perception of their own burnout status and as a result this is a target for further research.

Identifying protective and contributory factors may help to better serve doctors, patients and the community at large. It is the first step to inform appropriate interventions, which optimise the mental health of doctors and their overall functioning as well as improve patient care.^7^ Mental health care access needs to be opened up to this population, advertised and made easily accessible. Potential areas for intervention could include exercise programmes at work, strict capping of working hours, improving systemic problems such as the lack of resources and staff shortages.^4^ Addressing mental health education is also important as evidenced by the poor awareness of burnout difficulties among the sample population.

### Strengths

In terms of study strengths, this is the first of its kind in terms of a national study among community service doctors in SA. The largest South African study to focus on junior doctors was published more than 25 years ago, in 1994.^7^ This was prior to the requirement of a year of community service for South African medical graduates.^28^ Furthermore, it found a potentially novel hypothesis: that doctors may not be aware of their own burnout status.

### Limitations

The main limitation is the use of convenience sampling. Convenience sampling was used because of the difficulty in identifying the members of the study population at a national level. However, this leads to the likelihood of selection and sampling bias. Females were overrepresented in the sample population and female gender is likely to be a contributory factor to burnout.^13^ Those who are burnt out may be more likely to answer a survey on well-being. However, attempts were made to minimise this by not mentioning ‘burnout’ in the questionnaire (aside from the final question), the consent information referred to a ‘well-being survey’ instead, as instructed by the MBI manual. Over-representation of Mpumalanga province and under-representation of KZN was a further limitation. Electronic surveys among medical doctors generally yield low response rates, being another limiting factor.^30^ However, the calculated sample size was obtained. The results therefore should be interpreted with caution and may overstate the prevalence of burnout because of the likely selection bias inherent in the study design. Nevertheless, the results are valuable in drawing attention to the well-being of community service doctors in a national sample.

### Implications

If it is not recognised, burnout among doctors has a number of consequences for patient care, doctor mental and overall health and quality of life, as well as society as a whole.^1,3,9,15,16,24^ The majority of community service doctors are burnt out. A significant number of those who are burnt out want to leave the government sector, indicating that staff well-being is important to retain human resources in the state sector. Cognisance of factors that contribute to and protect against burnout is integral to improving the mental health of doctors and the overall care of patients.

The relationship between burnout and depression requires further research, as this study merely screened for major depression and did not use a diagnostic tool. This may guide identification and treatment strategies for burnout. Qualitative research into the nuances of the causes of burnout would also be helpful in elucidating further potential contributory and protective factors. More detailed research on outcomes of the burnt out doctor on patients would also be useful. Further studies on doctor’s self-awareness of burnout are required and the impact on health-seeking behaviour and other consequences thereof. Further studies will be required using probability sampling to confirm the results of this study.

## Conclusion

A large proportion of community service doctors in SA experience symptoms of burnout and a significant proportion are unaware of their burnt out status. There are a number of modifiable contributory and protective factors identified that may be targets for future mental health interventions.
